# Domestic Summary

**Published:** 1839-03-30

**Authors:** 


### DOMESTIC SUMMARY.

*Louisville Medical Journal.*—We regret to learn that the publication of this periodical has been suspended, no number having appeared since the second.

